# Spatial-temporal clusters of host-seeking *Aedes albopictus*, *Aedes japonicus*, and *Aedes triseriatus* collections in a La Crosse virus endemic county (Knox County, Tennessee, USA)

**DOI:** 10.1371/journal.pone.0237322

**Published:** 2020-09-03

**Authors:** R. D. Rowe, A. Odoi, D. Paulsen, A. C. Moncayo, R. T. Trout Fryxell

**Affiliations:** 1 Department of Entomology and Plant Pathology, University of Tennessee, Knoxville, Tennessee, United States of America; 2 Department of Biomedical and Diagnostic Services, University of Tennessee, Knoxville, Tennessee, United States of America; 3 Tennessee Department of Health, Nashville, Tennessee, United States of America; Faculty of Science, Ain Shams University (ASU), EGYPT

## Abstract

A bite from a La Crosse virus (LACV) infected *Aedes* mosquito can cause La Crosse encephalitis (LACE), which is a neuro-invasive disease that disproportionately affects children under the age of 16 in Southern Appalachia. The three vectors for LACV are *Aedes albopictus* (Skuse), *Ae*. *japonicus* (Theobald), and *Ae*. *triseriatus* (Say). Localized maps of the geographic distribution of vectors are practical tools for mosquito management personnel to target areas with high mosquito abundance. This study hypothesized that LACV vectors have unique species-specific spatial and temporal clusters. To test this, 44 sites were identified in Knox County, Tennessee for their land use/type. At each site, host-seeking mosquitoes were collected approximately every other week from May-October 2018. Spatial clusters of host-seeking mosquito collections for each of the three mosquito species were investigated using Kulldorff’s spatial scan statistic, specifying a retrospective space-time Bernoulli model. Most vector clusters were identified in south-central Knox County while the seasonality of clusters varied by mosquito species. Clusters of *Ae*. *albopictus* were observed throughout the entire study period while clusters of *Ae*. *japonicus* and *Ae*. *triseriatus* only occurred May-June. The findings indicate that the relative abundance of LACV vectors were more abundant in south-central Knox County compared to the rest of the county. Of interest, these clusters spatially overlapped with previous LACE diagnosed cases. These findings are useful in guiding decisions on targeted mosquito control in Knox County and may be applied to other counties within Southern Appalachia.

## Introduction

La Crosse virus (LACV) remains a persistent arboviral threat in the Southern Appalachian region of the United States (US) since pediatric encephalitis cases caused by this pathogen emerged in Southern Appalachia in the 1990s [1]. Infection with LACV may lead to La Crosse Encephalitis (LACE), a neuroinvasive disease that disproportionately affects children under the age of 16 [2]. Individuals infected with LACV may be asymptomatic or symptomatic with fever, brain swelling, potential cognitive damage and, although rare, death [3].

LACV is maintained in a zoonotic cycle between *Ae*. *triseriatus* (Say) and sciurid rodent reservoirs, as well as through transovarial transmission from infected female to offspring [4,5]. Proposed LACV bridge vectors include *Ae*. *albopictus* (Skuse) and *Ae*. *japonicus* (Theobold) [6–11], which was further speculated with the discoveries of established *Ae*. *albopictus* [12] *Ae*. *japonicus* populations [13] in 2003 and the simultaneous increase of LACE cases from less than two a year to an average of 20 cases a year in southern Appalachia [1]. Studies following the Tennessee outbreak indicated that LACE sites had *Ae*. *triseriatus* populations and larger *Ae*. *albopictus* populations [14,15]. The densities of these mosquitoes often vary throughout the season with *Ae*. *triseriatus* populations emerging in May and peaking in June, while *Ae*. *albopictus* populations emerge in June and peak in August / September; *Ae*. *japonicus* populations are still poorly understood in Tennessee [16–19]. The density of *Ae*. *albopictus* to *Ae*. *triseriatus* mosquitoes in a single mixed-forested hardwood setting was described as 1.2:1 [16], and we reported varying densities at additional Knox county sites which included previously LACV-positive homes [18,19]. Combined, this indicates that the *Aedes* populations are heterogeneous across space and time making targeted control difficult without surveillance.

One potential way to lower the incidence of LACE is to identify and visualize areas that have relatively more LACV vectors compared to other sites (or what is expected). Health professionals and pest management experts could then use the generated maps of LACV vector relative abundance sites to assist in related vector control programs. Without LACV vectors, LACV transmission is nearly impossible, thus managing vectors will very likely lower the incidence of LACE. We used Kulldorff’s spatial-scan statistic to statistically analyze and create these cluster maps [20]. Kulldorff’s scan statistic has successfully been used and implemented in mosquito control for both vector and disease predictions. For example, clusters of malaria vectors in the larval stage were identified in northern Sudan using this method [21]. This method was also used to identify clusters of West Nile encephalitis incidence throughout the United States [22] and proactively predict potential areas at-risk for West Nile virus through the identification of clusters of dead birds in New York [23]. In East Tennessee, this scan statistic was used to identify clusters of LACV vectors where clusters of *Ae*. *triseriatus* and *Ae*. *albopictus* overlapped the home of a fatal LACE case and locations where LACV vector mosquito pools were PCR positive for LACV [24].

The objective of this study was to identify statistically significant geographic and temporal clusters of LACV vector relative abundance in Knox County, Tennessee where approximately five LACE pediatric cases occur each year in the county. We tested the hypothesis that the three *Aedes* species have unique species-specific spatial and temporal patterns. We expect the findings from this study to provide useful information for guiding targeted vector control for county health departments, mosquito control, and state health departments; as well as areas for increased epidemiological studies on LACV.

## Materials and methods

### Study area

The study was conducted in Knox County, Tennessee; a county within the eastern region of the state that was selected because it is endemic with LACE [2,25]. The county has a heterogeneous landscape with Knoxville serving as the largest city surrounded by smaller towns, farmlands (e.g., crop production, horse stables, cow/calf operations), and urban and rural forested areas. Based on NOAA’s 1981–2010 three-decade averages calculated with weather data from Tyson Mcghee Airport, a Knox County airport, the general average temperature in Knox County is 25.05°C in the summer and 4.66°C in the winter. The average summer precipitation in Knox County is 40.48cm and the average winter precipitation is 33.22cm [26].

### Site selections

**Fig 1:**
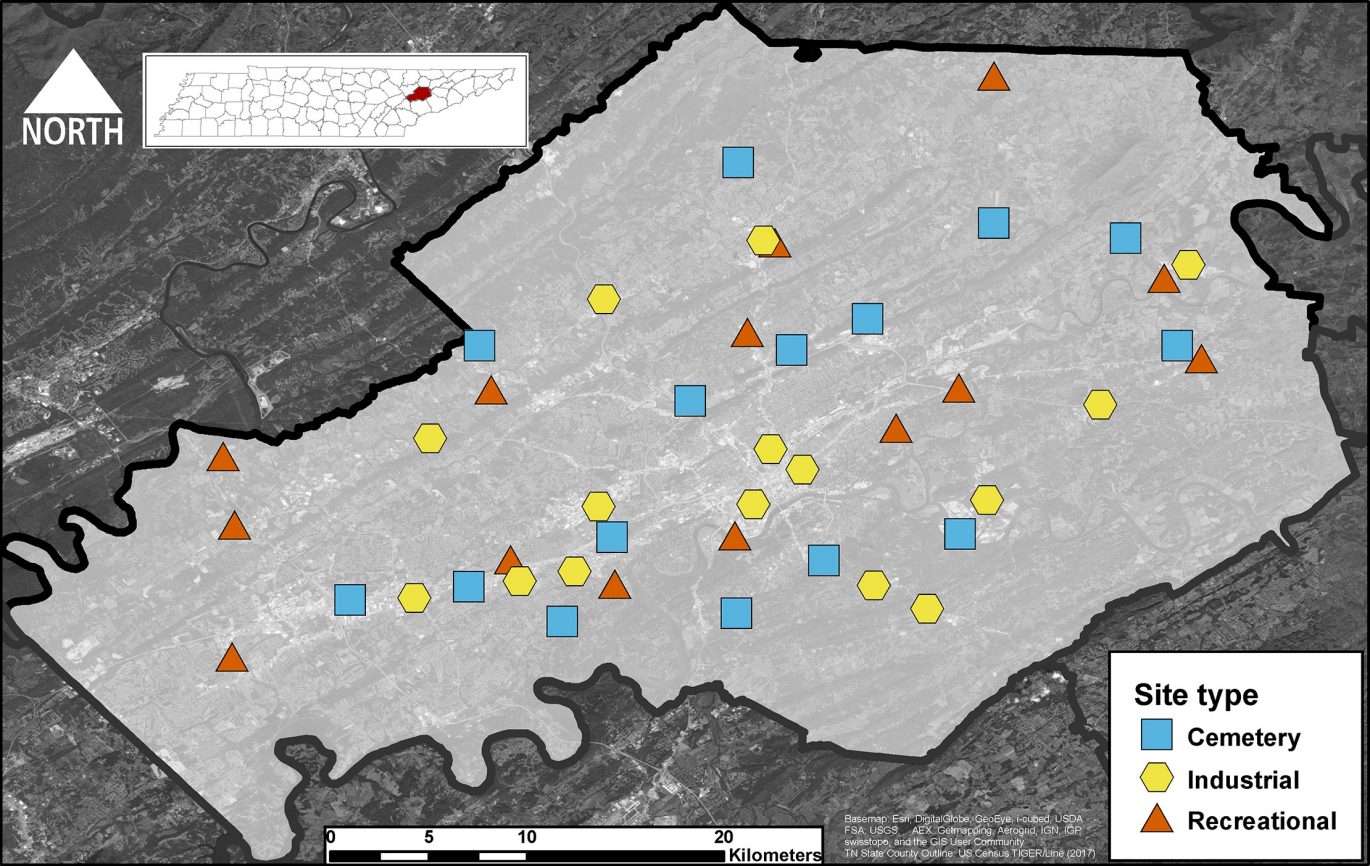
Study sites. Map of study area (Knox County, Tennessee), surrounding counties, and the 44 sites by site category. Content is the intellectual property of Esri and is used herein with permission. Copyright © 2019 Esri and its licensors. All rights reserved.

Mosquitoes were sampled from 44 unique publicly accessible sites within Knox County. Sites were characterized as either cemetery (n = 15), recreational (n = 14), or industrial (n = 15) sites. These site types were selected to devise a heterogenous gradient of areas that have abundant vegetation to areas with more impervious concrete surfaces. Cemetery sites were characterized with the inclusion of artificial containers in the form of vases and presence of at least 10 or more headstones. These sites were surrounded with tree lines and sometimes within forested areas. Recreational sites were either county-maintained parks or other outdoor recreational areas such as a garden or playground. These sites commonly had walking trails, athletic spaces (e.g., soccer field, baseball field), and/or presence of recreational equipment. The industrial sites were placed near commercial and industrial park areas and were characterized by general presence of land impervious structures such as concrete and large buildings (e.g., warehouse or hospital). All sites were at least 450 meters apart (mean distance of site to nearest site: 2.58 kilometers ± standard error 0.214) to prevent *Aedes* mosquito from one site overlapping with another (Fig 1). Previously, the average maximum distance of these vectors was calculated as 676m for *Ae*. *albopictus*, 362m for *Ae*. *triseriatus*, and 1600m for *Ae*. *japonicus* [27]; thus, most sites were distributed outside of the predicted average maximum distance for these species.

Geocoding of sites and calculation of distance between sites to their closest neighboring site was preformed using the ‘near’ tool in ArcMap 10.6.1(Environmental Systems Resource Institute, ArcMap 10.6.1 ESRI Redlands, CA). No specific permissions were required for sampling at the 44 sites as they were all open to the public; however, the county health department, parks and recreation, and local law enforcement were informed.

### Mosquito collections

Adult mosquitoes were collected twice a month, usually every other week, from May- October of 2018. A Center for Disease Control (CDC) light trap (model 512 John W. Hock, Gainesville, Florida) with the light removed was baited with ~1kg of dry ice inside a punctured sports cooler and a Biogents sweet scent lure (Biogents AG, Regensburg, Germany). The trap and bait combination has been shown to be an effective way to collect LACV vectors and removing the light is a way to eliminate by-catch or non-targets which can damage mosquitoes for identification [18]. These traps operated for ~24 hours in hidden and/or shaded areas of the site to ensure they were not stolen or disrupted by the public. Due to logistical limitations, trapping did not occur at all 44 sites on the same day. Instead, approximately half of the sites had a trap operating on one day and then the other half were trapped the next day. We minimized geographical bias by operating traps throughout the county and not operating traps in one region instead of the other (i.e. the entire county was sampled both trapping nights). Collections were brought back to the laboratory and identified within 48-hour time window post collection by paralyzing them with trimethylamine and then identifying adults to sex and species [28,29]. Specimens were stored at -80⁰C in pools of less than 15 mosquitoes consisting of the same species and sex and collected from the same site by each collection date. Mosquito pools were then screened for LACV at the Tennessee Department of Health. Viral RNA was extracted from each mosquito pool; pools were homogenized on a Retsch MM300 shaker for 90sec, centrifuged at 5000 rpm for 5 min, and then virus was isolated using the QIAamp viral isolation 96-welll protocol on the BioRobot 9604 or QIAamp Viral mini kit (Qiagen, Valencia, California). To detect LACV, 5μl of extract was then amplified using previously published protocols [30].LACV positive (LACV-positive mosquito pool and LACV isolate) and negative (*Culex* extracted RNA, water template) controls were used in all assays.

### Statistical analysis

Summary statistics for mosquito species were calculated in R statistical software using the package pastecs version 1.3.21 [31]. This R package was used in R studio version 1.1.463 [32] with R version 3.5.3 [33]. Data associated with instances of trap malfunctioning or tampering were removed from statistical analysis (n = 50 individual events). For three other collection events, two sites did not have traps set-up yet at the start of the study. They were subsequently added after the first collection week.

Retrospective space-time scan statistics, implemented in SaTScan version 9.6 (Martin Kulldorff, Boston, MA, US), were used to investigate and identify space-time clusters of mosquito abundance for each of the three-mosquito species. This analysis uses a circle or overlapping cylindrical window that moves across a map of specified longitude/latitude coordinates or centroids and expands and contracts based on the determined scanning window and probability distribution through space and/or time [34]. A retrospective analysis indicates the scan statistic was done in a fixed geographical region (Knox County) over a fixed study period (May-October 2018) [34]. The scanning window radii fluctuates during the scan and can vary from 0 to a user specified maximum value. To determine statistically significant clusters, a likelihood ratio test is performed, and Monte Carlo hypothesis testing is used to obtain *p-*values [34]. The null hypothesis of this scan statistic is that the relative abundance within the cluster is the same as outside of the cluster. The alternative hypothesis states that there exists at least one cluster with a different relative abundance, either higher or lower, within the window compared to the relative abundance outside of the window in a given area and in a given time [20]. The results of the likelihood ratio test provide cluster ranks that signify how likely the cluster occurred not by chance.

A Bernoulli probability distribution was used in this retrospective space-time model to identify spatial-temporal clusters of interest in areas with higher abundance of *Ae*. *albopictus*, *Ae*. *triseriatus*, or *Ae*. *japonicus* than expected. We used the Bernoulli probability distribution as it has been found to be effective in mosquito surveillance on the larval level [21] and it was previously used to identify significant spatial clusters of LACV vectors [24]. In the Bernoulli distribution, each species was classified as ‘cases’ and all other species were classified as a ‘controls’. Cases were defined for each collection session as the LACV vector of interest and controls were defined as the total number of mosquitoes collected in that event excluding the LACV vector of interest. For example, if one trap collected 100 mosquitoes and 60 were *Ae*. *albopictus*, then the case number for that event is 60 and the control number is 40.

For the spatial window of the scan statistic, two different retrospective space-time scans with two different maximum window sizes were used for each LACV vector, resulting in six independent spatial scan analyses. One spatial scan used a cylindrical spatial window where clusters were determined within a window through space and time that could expand and contract from values that ranged from 0 to a maximum of 50% of the mosquito population. These specifications have been found to identify high abundance areas of LACV vectors that overlapped with a fatal LACE case in a retrospective spatial only Bernoulli scan statistic [24,34]. The findings from the analyses with this window specification will be referred to as the study-wide scan results. The second scan used the same potential maximum window size of 50% of the mosquito population, but was constrained not to exceed one kilometer. This was done because most LACV vectors have a flight range that generally does not exceed one kilometer [35]. The results from the second scan window will be referred to as the site-specific scan. The purpose of these two scanning windows was to identify high abundance areas within Knox County based on specifications similar to past LACV vector cluster studies [24] as well as identify specific sites within the study area that have more LACV vectors based on mosquito biology. The temporal scanning windows for the study wide and site-specific scans were aggregated in 14-day intervals to match the adult collections that occurred approximately every two weeks. Monte Carlo hypothesis testing was used with 999 replications. Clusters with a *P*-value > 0.05 were considered non-significant and were not reported except one cluster that had a marginally significant *P*-values of 0.058. All statistically significant clusters were mapped using Arcmap 10.6.1 in a composite map and 10 individual maps were compiled in a 50 second video showing the spatial and temporal occurrences of clusters. This video was created in Movie Studio Platinum 15 (MAGIX, Berlin, Germany).

## Results

### Mosquito collections

A total of 6,739 adult mosquitoes were collected and this included six genera (*Aedes*, *Anopheles*, *Culex*, *Orthopodomyia*, *Psorophora*, and *Uranotaenia*) representing 20 species. All three LACV vectors were identified: *Ae*. *albopictus* (77.0% of all mosquitoes collected), *Ae*. *triseriatus* (4.0%), and *Ae*. *japonicus* (1.1%). Other species identified included 4.7% *Ae*. *vexans* (Meigen), 3.2% *Culex pipiens* complex, 1.9% *Cx*. *restuans* (Theobald), 1.8% *Cx*. *erraticus* (Dyan and Knab), and 1.5% *Anopheles punctipennis* (Say). The remaining 4.0% consisted of *Ae*. *trivittatus* (Coquillett), *Ae*. *tormentor* (Dyar and Knab), *An*. *quadrimaculatus* (Say), *Cx*. *territans* (Walker), *Cx*. *salinarius* (Coquillett), *Orthopodomyia signifera* (Coquillet), *Psorophora ciliata* (Fabricius), *Ps*. *columbiae* (Dyar and Knab), *Ps*. *cyanescens (*Coquillett), *Ps*. *ferox* (Von Humbolt), *Ps*. *howardii* (Coquillett), and *Uranotaenia sapphirina* (Osten and Sacken). Unfortunately, 137 specimens could not be identified to species because they had lost their morphological features for identification. These 137 specimens included 53 *Aedes* specimens, 73 *Culex* specimens, nine *Psorophora* specimens, and two *Anopheles* specimens (Table 1).

**Table 1:** Summary statistics of host-seeking mosquitoes collected around Knox County, Tennessee, with a CDC-light trap baited with dry ice and the BG-Biogents lure. La Crosse virus vectors bolded.

Species	Abundance (%)	Mean ± Standard Error	Median (Range)	No. Sites (%)	No. Weeks (%)
***Aedes albopictus***	**5239 (77.0%)**	**11.03 ± 1.657**	**3 (0–564)**	**44 (100%)**	**12 (100%)**
***Aedes triseriatus***	**271 (4.0%)**	**0.57 ± 0.139**	**0 (0–45)**	**24 (54.5%)**	**12 (100%)**
***Aedes japonicus***	**72 (1.1%)**	**0.15 ± 0.106**	**0 (0–50)**	**9 (20.5%)**	**9 (75%)**
*Aedes tormentor*	1 (0.01%)	0.002 ± 0.0021	0 (0–1)	1 (2.3%)	1 (8.3%)
*Aedes trivittatus*	13 (0.2%)	0.03 ± 0.012	0 (0–4)	8 (18.2%)	5 (41.7%)
*Aedes vexans*	318 (4.70%)	0.67 ± 0.084	0 (0–21)	36 (81.2%)	12 (100%)
Unknown *Aedes* species	53 (0.8%)	0.11 ± 0.032	0 (0–11)	17 (38.6%)	10 (83.3%)
*Anopheles punctipennis*	99 (1.5%)	0.21 ± 0.030	0 (0–6)	31 (70.5%)	12 (100%)
*Anopheles quadrimaculatus*	30 (0.4%)	0.06 ± 0.017	0 (0–6)	15 (34.1%)	10 (83.3%)
Unknown *Anopheles* species	2 (0.03%)	0.004 ± 0.0030	0 (0–1)	2 (4.5%)	2 (16.7%)
*Culex erraticus*	124 (1.8%)	0.26 ± 0.066	0 (0–23)	25 (56.8%)	9 (75%)
*Culex pipiens* complex	219 (3.20%)	0.460 ± 0.0801	0 (0–16)	29 (65.9%)	12 (100%)
*Culex restuans*	126 (1.9%)	0.265 ± 0.0450	0 (0–13)	26 (59.1%)	11 (91.7%)
*Culex salinarius*	6 (0.09%)	0.013 ± 0.0089	0 (0–3)	2 (4.5%)	1 (8.3%)
*Culex territans*	29 (0.4%)	0.061 ± 0.0235	0 (0–8)	9 (20.1%)	6 (50%)
Unknown *Culex* species	73 (1.1%)	0.153 ± 0.0529	0 (0–23)	22 (50%)	11 (91.7%)
*Orthopodomyia signifera*	4 (0.06%)	0.008 ± 0.0066	0 (0–3)	2 (4.5%)	2 (16.7%)
*Psorophora ciliate*	3 (0.04%)	0.006 ± 0.0036	0 (0–1)	3 (6.8%)	3 (25%)
*Psorophora columbiae*	15 (0.2%)	0.031 ± 0.0010	0 (0–3)	8 (18.2%)	5 (41.7%)
*Psorophora cyanescens*	2 (0.03%)	0.004 ± 0.0030	0 (0–1)	2 (4.5%)	1 (8.3%)
*Psorophora ferox*	21 (0.3%)	0.044 ± 0.0242	0 (0–11)	5 (11.4%)	8 (66.7%)
*Psorophora howardii*	3 (0.04%)	0.006 ± 0.0047	0 (0–2)	2 (4.5%)	2 (16.7%)
Unknown *Psorophora* species	9 (0.1%)	0.02 ± 0.006	0 (0–1)	9 (20.1%)	7 (58.3%)
*Uranotaenia sapphirina*	7 (0.1%)	0.02 ± 0.006	0 (0–2)	6 (13.6%)	4 (33.3%)
**Total**	**6739**	**14.16 ± 1.821**	**5 (0–602)**	**44 (100%)**	**12 (100%)**

### LACV vector relative abundance

For the LACV vectors, there was a total of 5,239 *Ae*. *albopictus* to 1,500 controls, 271 *Ae*. *triseriatus* to 6468 controls, and 72 *Ae*. *japonicus* to 6667 controls. As the mosquito populations changed over time, the controls for each species differed by calendar week (Table 2). A total of 841 LACV pools were screened for LACV, and these consisted of 745 *Ae*. *albopictus* pools (mean of 7.4 mosquitoes per pool), 74 *Ae*. *triseriatus* pools (mean of 3.7 mosquitoes per pool), and 22 *Ae*. *japonicus* pools (mean of 3.4 mosquitoes per pool). All of the pools were LACV-negative.

**Table 2:** Mosquito cases and controls. Mosquito case (LACV vector species) and control (total mosquitoes excluding vector case species) data for the three study LACV vectors.

2018 Calendar Week	No. Sites ^a^	*Aedes albopictus*		*Aedes triseriatus*		*Aedes japonicus*		Total
		Cases	Controls	Cases	Controls	Cases	Controls	
20	39	41	96	26	111	3	134	137
23	34	155	130	59	226	10	275	285
24	38	278	188	54	412	51	415	466
26	39	130	31	13	148	0	161	161
28	41	411	100	25	486	1	510	511
30	41	461	70	10	521	1	530	531
33	41	723	161	24	860	0	884	884
34	42	266	102	10	358	0	368	368
36	40	889	171	30	1030	1	1059	1060
38	42	1068	172	14	1226	2	1238	1240
40	40	557	117	5	669	2	672	674
42	38	260	162	1	421	1	421	422

^a^ Due to trap malfunctions, tampering, or site not being set-up, not all sites operated simultaneously.

### *Aedes albopictus s*patial-temporal clusters

The study-wide spatial scan of *Ae*. *albopictus* identified four statistically significant clusters. The cluster with the lowest *P-*value was comprised of six sites and occurred from August 11 to October 5. This cluster had 1641 *Ae*. *albopictus* when 1402 mosquitoes were expected (risk ratio = 1.25; *P* < 0.001). The second cluster with lowest *P-*value occurred between June 30 and September 7 and four sites occurred inside the cluster. This cluster had an observed 557 *Ae*. *albopictus* when 456 mosquitoes were expected (risk ratio = 1.25; *P* < 0.001). The third cluster was from June 30 to September 7 and it had one site with an observed 103 *Ae*. *albopictus* when 81 were expected (risk ratio = 1.28, *P* < 0.001). The fourth cluster was from July 14 to September 21 and it had four sites, which included 119 *Ae*. *albopictus* observed when 96 mosquitoes were expected (risk ratio = 1.25; *P* < 0.001).

**Fig 2:**
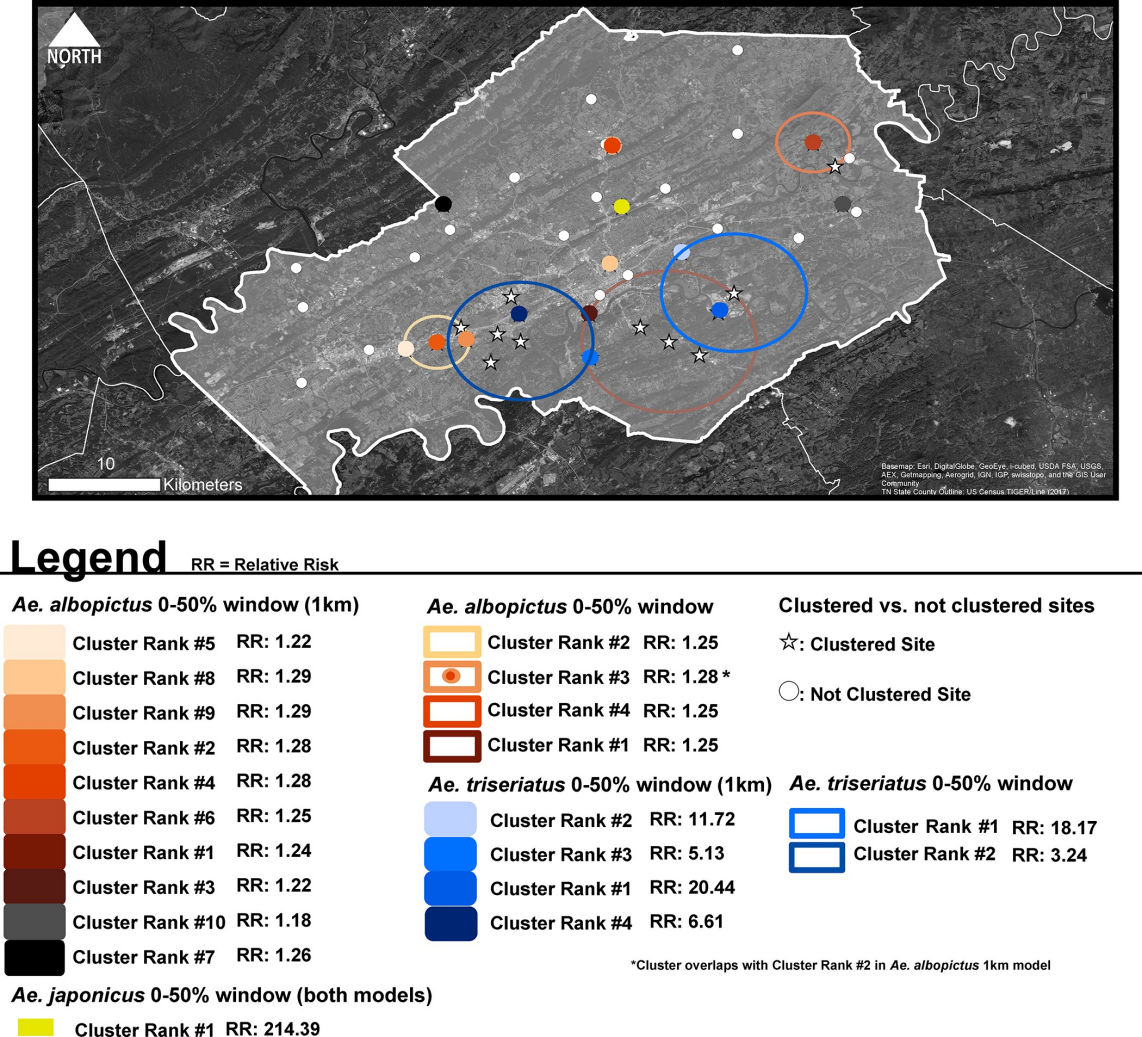
Composite cluster map. Composite image of SatScan results of the 3 LACV vectors within Knox County Tennessee, May-October 2018. Content is the intellectual property of Esri and is used herein with permission. Copyright © 2019 Esri and its licensors. All rights reserved.

The site-specific *Ae*. *albopictus* spatial scan resulted in ten statistically significant clusters throughout Knox County that had higher counts of *Ae*. *albopictus* than expected by the spatial scan statistic. Of the ten sites, seven were identified within clusters previously identified in the study-wide scan and the other three sites were at sites that were not within the clusters identified in the study-wide scan. All ten *Ae*. *albopictus* clusters occurred at different times within the study period. Two site-specific clusters started in May, three site-specific clusters started in June, two clusters started in July, and three site-specific clusters occurred in August. All site-specific clusters had risk ratios that spanned from 1.18–1.29 (*p* < 0.05). Specific statistical details of the site-specific clusters, as well as the four clusters identified in the study wide spatial scan, are detailed in the maps and tables (Fig 2, S1 Movie, Tables 3 and 4).

**Table 3:** Study-wide spatial scan results. Statistical results of the study-wide spatial scan for *Aedes albopictus*, *Ae*. *triseriatus*, and *Ae*. *japonicus*. Reported are the number of sites in clusters, date of cluster, the ratio between observed/expected mosquitoes, and the relative risk with the corresponding *P-*value from Monte Carlo hypothesis testing. Table is sorted by date of cluster.

Species	Cluster Rank	No. Sites In Cluster	Cluster Dates	Mosquito Ratio	Relative Risk (P-value)
*Aedes albopictus*	2	4	30 June– 7 September	1.22	1.25 (*P* < 0.001)
	3	1	30 June– 7 September	1.27	1.28 (*P* < 0.001)
	4	2	14 July– 21 September	1.24	1.25 (*P* < 0.001)
	1	6	11 August– 5 October	1.17	1.25 (*P* < 0.001)
*Aedes triseriatus*	1	3	16 May– 29 June	11.64	18.17 (*P* < 0.001)
	2	8	16 May– 29 June	3.09	3.24 (*P =* 0.026)
*Aedes japonicus*	1	1	2 June– 15 June	54.35	214.39 (*P* < 0.001)

**Table 4:** Site-specific spatial scan results. Statistical results of the site-specific spatial scan for *Aedes albopictus*, *Ae*. *triseriatus*, and *Ae*. *japonicus*. Statistical results reported are the date of cluster, Fig 2 associated maps, the ratio between observed/expected mosquitoes, and the relative risk with the corresponding *P-*value from Monte Carlo hypothesis testing. Table is sorted by date of cluster.

Cluster Rank	Cluster Dates	Mosquito Ratio	Relative Risk (P-value)
***Aedes albopictus***			
5	16 May– 27 July	1.21	1.22 (*P* < 0.001)
8	16 May– 27 July	1.29	1.29 (*P* < 0.001)
9	16 June– 24 August	1.29	1.29 (*P* = 0.003)
2	30 June– 7 September	1.26	1.28 (*P* < 0.001)
4	30 June– 7 September	1.27	1.28 (*P* < 0.001)
6	14 July– 21 September	1.25	1.25 (*P* < 0.001)
1	14 July– 21 September	1.18	1.24 (*P* < 0.001)
10	11 August– 5 October	1.17	1.18 (*P =* 0.015)
7	11 August– 21 September	1.25	1.26 (*P* < 0.001)
3	25 August– 5 October	1.21	1.22 (*P* < 0.001)
***Aedes triseriatus***			
2	16 May– 29 June	9.43	11.72 (*P* < 0.001)
3	16 May– 29 June	4.97	5.13 (*P* = 0.008)
1	2 June– 15 June	17.22	20.44 (*P* < 0.001)
4	2 June– 29 June	6.49	6.61 (*P* = 0.058)
***Aedes japonicus***			
1	2 June– 15 June	54.35	214.39 (*P* < 0.001)

### *Aedes triseriatus* spatial-temporal clusters

Two clusters were identified in the study wide spatial scan of *Ae*. *triseriatus*. The primary cluster with the lowest *P-*value, consisting of three sites, was detected between May 16 to June 29 and had 103 *Ae*. *triseriatus* when nine *Ae*. *triseriatus* were expected (risk ratio = 18.17; *P* < 0.001). The secondary cluster, comprised of eight sites, occurred from May 16 to June 29, and had 18 *Ae*. *triseriatus* observed when six *Ae*. *triseriatus* were collected (risk ratio = 3.24; *P* = 0.026). In the site-specific spatial scan, there were four clustered sites, and all were present within the study wide scan of *Ae*. *triseriatus*. These clusters were early in the season and included two from May 16 to June 29 and two from June 2 to June 29 (Fig 2, S1 Movie, Tables 3 and 4).

### *Aedes japonicus* spatial-temporal clusters

Only 72 *Ae*. *japonicus* were collected during the study and 80.6% of these were collected from one site (Fig 2, S1 Movie, Tables 3 and 4). The cluster from both spatial scans ranged from June 2 to June 15 and it included 54 *Ae*. *japonicus* collected when only one mosquito was expected (risk ratio = 214.39; *P* < 0.001).

## Discussion

Data presented here supports our hypothesis that the three *Aedes* species have unique species-specific spatial and temporal distributions. Throughout the entire study, *Ae*. *albopictus* clusters were pervasive. Clusters of *Ae*. *triseriatus* were specific such that both the study wide and site-specific clusters were concentrated in the southern area of the county from the middle of May to the end of June. There was only one site that had more *Ae*. *japonicus* than expected and this single cemetery site was noted for its dense canopy coverage next to the cemetery. Although this study was not investigating environmental factors, other studies have detailed the importance of forested environments for *Ae*. *japonicus* [36,37]. Similar clustering patterns were previously reported of LACV vectors in Tennessee where *Ae*. *albopictus* had two clusters, *Ae*. *triseriatus* followed with a single cluster, and *Ae*. *japonicus* was rarely collected and did not present any clustering [24]. The distribution of the clusters may be explained by the variation in distributions of the combination of abiotic, biotic, and/or socioeconomic predictors. If there is localized spatial variation of LACV vector mosquito populations in the study area, then it is likely there is localized environmental variation that generates areas with increased mosquito populations. While this study did not address these questions, future studies should.

Additionally, we screened pools of these mosquitoes by site and date for LACV and all mosquitoes were negative for LACV; however, we do not know if these negative samples were truly negative or due to multiple freezer malfunctioning events. Knowing where these mosquitoes are occurring more often than normal, we can use these results in future studies to investigate the spatial-temporal incidence of LACV in both the vectors and their reservoir hosts. Discovering spatial-temporal distributions of LACV vectors is important as it provides insight into where LACV transmission may occur. For example, using LACV-mosquito data associated with a LACE fatality [38] in a purely spatial Bernoulli scan statistic allowed researchers to identify that the overlap of *Ae*. *albopictus* and *Ae*. *triseriatus* clusters was also associated with LACV-infected mosquitoes and the fatal case [24].

The CDC reports LACE cases occur from June through September [39], with an intrinsic incubation period of 5–15 days [40]. We compared these cluster distributions with the temporal patterns of LACE cases and found that *Ae*. *triseriatus* clusters before and *Ae*. *albopictus* clusters before and after the average temporal window of LACE cases; thus, we speculate that these clustering events could serve as predictors for LACV transmission windows. During this study, the Knox County health department reported three LACE cases. Two cases were not in our spatial clusters, but one case was within the spatial intersection between a detected *Ae*. *albopictus* and *Ae*. *triseriatus* cluster. We subsequently screened our collections of LACV vectors found at the LACE case houses and within LACV clusters with the Tennessee Department of Health and found none of the host-seeking mosquitoes were LACV positive. It is unfortunate to this study that all of the mosquito pools were LACV negative.

Additionally, there are spatial similarities between the clusters presented in this study to past LACE epidemiological investigations in the region. Confirmed LACE pediatric cases from 1997 to 2006 were analyzed at the census tract level to identify spatial clusters in eastern Tennessee with a Local Indicator of Spatial Autocorrelation (LISA) analysis; the results indicated the incidence of both high- and low-risk LACE clusters occurred in central and south-central of census tracts within Knox county [25]. Those same LACE risk census overlapped with our study wide vector clusters. In a blinded-cohort study following LACE case and non-case houses in east Tennessee identified that the abundance of *Ae*. *albopictus* larvae and adults were three times greater at case houses compared to non-case houses [14]. While there is no direct link between those studies and our results, the spatial overlaps from the two independent analyses, previous confirmation of spatially associated LACE cases in North Carolina [41], and evidence that abundance of *Ae*. *albopictus* may increase the risk for LACE cases in east Tennessee provides an interesting insight and potential lead for future studies.

As with all statistical analyses, there are limitations to what the analysis can and cannot tell us about vector populations within an area. A caveat in this study was using the Bernoulli probability distribution because this distribution compares cases (species) to the control (other species) and that this distribution assumes that all mosquitoes behave similarly. An alternative approach is to run a scan statistic with the Poisson model, which assumes data follow a Poisson distribution; however, data here were over dispersed and the mean and variance of all mosquitoes were not similar. There are methods to run spatial-scan statistics using an over-dispersed non-normal probability distribution like a negative binomial [42,43]; however, software to conventionally run those spatial statistics like SaTScan currently do not exist.

As this study focused on vectors of LACV and not the pathogen, information on LACV-infected mosquitoes would provide extra insight into these clusters. The current state of the analysis retrospectively shows time and sites where clustering of LACV vector mosquitoes were evident. In future studies, a prospective analysis could be considered. Unlike the retrospective analysis, prospective analysis is used for early detection of clusters [22]. Reliability and validity of a prospective analysis at predicting areas with more LACV vectors and/or potentially infected vectors is currently unknown.

Results of this study provide insight into the spatial and temporal patterns for *Aedes* vectors of LACV in Knox County, TN. The south-central region of Knox County should be considered for future LACV mosquito control research efforts. The results from this study, as well as previous investigations of LACE around and within Knox County, underscores the need for localized mosquito control because the damaging and lasting effects of LACE in children, which include cognitive abnormalities, should not be neglected.

## Supporting information

S1 Appendix (XLSX)

S1 File (ZIP)

S1 Movie Temporal and spatial clusters of the three LACV vectors through the 2018 study period. “Content is the intellectual property of Esri and is used herein with permission. Copyright © 2019 Esri and its licensors. All rights reserved.”
(MP4)
